# The rudimentary left ventricle does not impact on right ventricular size and function in hypoplastic left heart syndrome during serial follow up after Fontan completion

**DOI:** 10.1016/j.ijcchd.2025.100627

**Published:** 2025-10-09

**Authors:** Abigail Burleigh, Dominik Daniel Gabbert, Yujiro Ide, Anselm Uebing, Inga Voges

**Affiliations:** a Department of Congenital Heart Disease and Paediatric Cardiology, University Hospital Schleswig-Holstein, Campus, Kiel, Germany; b German Centre for Cardiovascular Research, Partner Site Hamburg/Kiel/Lübeck, Kiel, Germany

**Keywords:** Hypoplastic left heart syndrome, Right ventricular volumes, Right ventricular function, Cardiovascular magnetic resonance

## Abstract

**Background:**

Previous studies in patients with hypoplastic left heart syndrome (HLHS) suggested that a larger left ventricle (LV) might negatively impact right ventricular (RV) function. This study aimed to analyse the impact of the presence of an LV remnant on RV size and function during serial follow up.

**Methods:**

Serial cardiovascular magnetic resonance (CMR) examinations after completion of the total cavopulmonary connection were retrospectively analysed. Patients were divided into those with and those without a rudimentary LV. RV and LV end diastolic and end systolic volumes as well as stroke volume, ejection fraction (RVEF, LVEF) and end diastolic mass were measured.

**Results:**

90 HLHS patients (female: 26) who had at least two CMR examinations were included. 51 patients had three examinations. 56 patients had an LV remnant, 34 did not. RV volumes and mass indexed to body surface area as well as RVEF did not differ significantly between both groups. LV volumes showed no association with RV volumes and RVEF.

**Conclusion:**

Analysis of serial CMR examination suggests that the presence of an LV remnant does not have a major impact on RV size and function during longer-term follow-up. Future studies might focus on regional RV function.

## 1 Introduction

Hypoplastic left heart syndrome (HLHS) is characterised by a complex spectrum of cardiac anomalies and can be divided into anatomical subtypes, all of which include a substantial hypoplasia of the left ventricle (LV), its associated structures and the ascending aorta, but are differentiated by the patency of the mitral and aortic valve [1].

Left ventricular size varies with the patency of the left sided valves and thus with the anatomic subtype [2]. Previous studies in HLHS patients have suggested that a larger or hypertrophied LV may impact negatively on right ventricular (RV) size and function [[3], [4], [5], [6]]. With the RV being the single and systemic ventricle in HLHS patients with Fontan circulation, this is of particular clinical interest as its function is one of the main determinants of long-term outcome [7], but only few studies that examined the impact of the presence or absence of a rudimentary LV on RV function by analysing longitudinal imaging data exist [4].

In this study, we hypothesise that RV volumes and RV ejection fraction (RVEF) differ between HLHS patients with and without an LV remnant. To test this hypothesis, serial RV volumes, RV mass and RVEF in both groups were measured using cardiovascular magnetic resonance (CMR).

## 2 Methods

### 2.1 Patients

HLHS patients after completion of the total cavopulmonary connection (TCPC) were retrospectively included. Inclusion criteria were [1] a minimum of two CMR examinations after completion of the TCPC [2], availability of short axis cine images and [3] the examinations had to be of sufficient quality. Patients with only one, incomplete or insufficient CMR examinations as well as patients with contraindications for CMR were excluded.

**Fig. 1:**
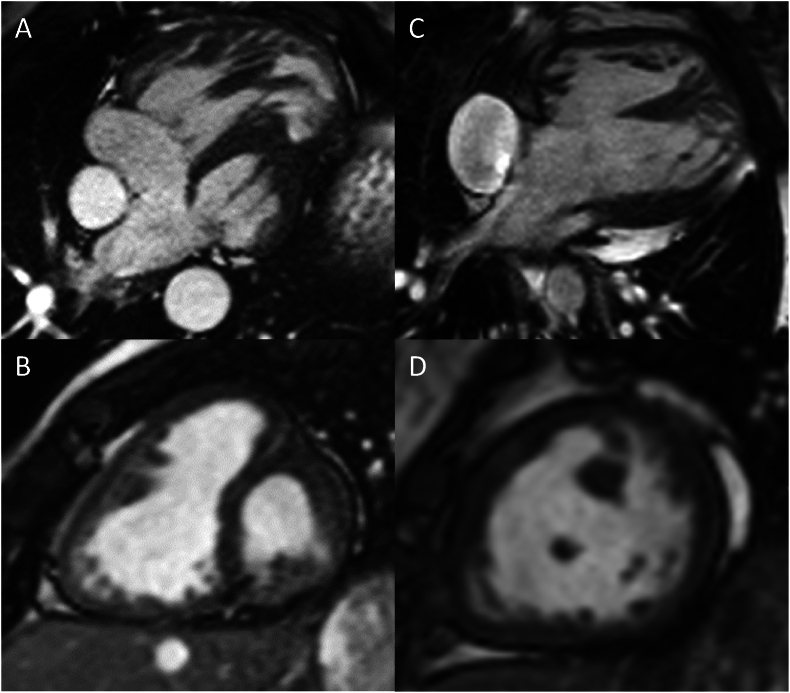
A, B) CMR 4-chamber- and short-axis views of a patient with HLHS and an LV remnant, C, D) the same views in a patient without an LV remnant.

Patients were divided into those with and those without an LV remnant (Fig. 1). The presence of a more than slit-like LV was defined as LV presence. CMR examinations and data from medical records were used to define the anatomical subtype in each patient.

Informed consent was obtained from the parents or guardians of the children enrolled into the study. The study protocol conforms to the ethical guidelines of the 1975 Declaration of Helsinki as reflected in a priori approval by the institution's ethics committee (ID Nr.: D503/20, date of approval June 12, 2021 and approval of amendment October 18, 2021).

### 2.2 Cardiovascular magnetic resonance

CMR studies were performed using a 1.5 T (T) or 3T scanner. For image postprocessing commercially available CMR software (cvi42 for Cardiovascular MRI, Circle Cardiovascular Imaging, Calgary, Canada; Medis Suite Solutions, Medical Imaging Software, Leiden, the Netherlands) was used.

Manual tracing of endo- and epicardial contours on short axis cine images was performed to assess RV end diastolic, end systolic and stroke volumes (RVEDV, RVESV), as well as RVEF and RV end diastolic mass (RVEDMM). Furthermore, volumes and EF of the rudimentary LV were measured. To achieve comparability, RV and LV volumes and mass were indexed to body surface area (BSA, RVEDVi, RVESVi, RVSVi, RVEDMMi, LVEDVi, LVESVi, LVSVi, LVMMi).

Two-dimensional (2D) phase-contrast images at the level of the distal neo-aortic anastomosis were used to measure peak velocity at the distal anastomosis.

### 2.3 Statistical analysis

Statistical analysis was performed using MedCalc Version 22.021. The Shapiro-Wilk test was used to assess normal distribution. As most data were not normally distributed, data are shown as median and 1st and 3rd quartiles (IQR) or median and range. Differences in RV volumetric parameters across both groups at the three different time points were analysed using the Mann-Whitney-U-Test. Spearman rank correlation was used to analyse associations between parameters. Adjustments for multiple testing were performed, and the significant p-value was reduced to 0.00033.

## 3 Results

90 patients after TCPC completion were included (female: 26; median age at TCPC: 2.6 years (y), range 1.1–5.4 y). All patients had at least two and 51 patients had three serial CMR examinations. Median interval between first and second examination was 5.3 y and 10.1 years between the first and third examination. The first examination was performed at a median age of 4.4 (2.3–11.5) y. Patients were classified by presence or absence of an LV remnant. 56 patients had an LV remnant, 34 did not.

Anthropometric characteristics and CMR results for the entire cohort are shown in Table 1.

**Table 1:** Patient characteristics and CMR results.

Parameters	1st examination (n = 90)	2nd examination (n = 90)	3rd examination (n = 51)
**Age (y)**	4.4 [3.7; 6.1]	10.0 [9,0; 11.7]	15.2 [13.5; 16.7]
**Weight (kg)**	16.7 [15.0; 19.0)	30.0 [25.0; 37.0]	51.0 [43.4; 63.5]
**Height (cm)**	104.0 [99.0; 111.0]	137.0 [128.8; 147.2]	162.0 [153.0; 172.5]
**BMI (kg/m^2^)**	15.5 [14.5; 16.4]	16.2 [15.1; 18.4]	19.5 [17.2; 23.0]
**BSA (m^2^)**	0.7 [0.6; 0.8]	1.1 [1.0; 1.3]	1.5 [1.4; 1.7]
**RVEDV (ml)**	62.1 [51.2; 72.4]	102.8 [83.1; 131.1]	159.5 [136.1; 198.5]
**RVESV (ml)**	28.1 [21.1; 35.1]	49.6 [36.8; 63.7]	80.0 [60.7; 100.9]
**RVSV (ml)**	32.0 [29.5; 37.8]	51.8 [45.9; 69.0]	79.5 [64.9; 93.7]
**RVEF (%)**	55.4 [50.2; 59.9]	54.2 [48.5; 58.9]	50.6 [45.0; 56.8]
**RVEDMM (g)**	34.5 [28.7; 46.4]	57.1 [40.5; 71.0]	68.6 [60.6; 92.1]
**RVEDVi (ml/ml^2^)**	85.7 [73.7; 97.2]	90.4 [74.4; 110.2]	107.4 [92.5; 118.8]
**RVESVi (ml/m^2^)**	37,0 [29.9; 46.8]	43.3 [34.4; 55.2]	52.0 [41.8; 66.8]
**RVSVi (ml/m^2^)**	47.3 [41.9; 52.4]	50.0 [43.0; 58.7]	53.3 [46.5; 60.3]
**RVEDMMi (g/m^2^)**	49.8 [39.2; 56.6]	49.3 [42.6; 59.8]	48.6 [41.1; 57.6]

BMI, body mass index; BSA, body surface area; RVEDV, right ventricular end diastolic volume; RVEDVi, BSA indexed right ventricular end diastolic volume; RVESV, right ventricular end systolic volume; RVESVi, BSA indexed right ventricular end systolic volume; RVSV, right ventricular stroke volume; RVSVi, BSA indexed right ventricular stroke volume; RVEDMM, right ventricular end diastolic mass; RVEDMMi, indexed right ventricular end diastolic mass.

Values shown as median with interquartile range.

Comparing both groups, RVEF, RVESVi, RVEDVi and RVEDMMi did not differ significantly at all three examinations (Table 2). LVEDVi and LVESVi did not correlate with RV volumes and ejection fraction (p < 0.05). Volumetrics for the LV remnant are shown in Table 3. There was also no significant association between RVEF and peak velocity at the distal anastomosis of the reconstructed aortic arch (p < 0.05).

**Table 2:** CMR results for patients with and without an LV remnant.

	1st examination		2nd examination		3rd examination	
Parameters	LV remnant (n = 56)	no LV remnant (n = 34)	LV remnant (n = 53)	no LV remnant (n = 34)	LV remnant (n = 30)	no LV remnant (n = 21)
**RVEDV (ml)**	62.0 [49.2; 73.1]	64.31 [53.4; 70.0]	102.4 [76.0; 131.9]	106.6 [93.0; 127.6]	162.0 [129.6; 204.5]	159.5 [141.3; 188.9]
**RVESV (ml)**	26.8 [20.6; 35.5]	30.0 [21.9; 34.2]	48.0 [33.0; 62.5]	51.8 [40.2; 66.3]	80.5 [52.1; 119.8]	77.4 [66.8; 93.2]
**RVSV (ml)**	31.6 [28.6; 41.2]	32.4 [31.0; 35.9]	49.8 [44.6; 71.8]	53.4 [46.4; 64.3]	78.7 [64.6; 95.5]	79.8 [72.3; 87.9]
**RVEF (%)**	56.8 [50.1; 60.7]	53.4 [50.4; 58.7]	54.6 [48.8; 59.0]	53.4 [47.6; 56.5]	51.4 [44.9; 59.3]	49.7 [45.2; 55.1]
**RVEDVi (ml/ml^2^)**	84.8 [72.6; 94.6]	87.8 [76.9; 100.0]	89.4 [76.4; 106.4]	94.6 [74.2; 111.7]	104.7 [88.2; 121.6]	107.9 [99.1; 115.8]
**RVESVi (ml/m^2^)**	35.0 [29.0; 45.1]	41.1 [32.7; 48.7]	39.6 [33.9; 51.9]	47.5 [37.0; 57.4]	50.5 [37.3; 67.4]	54.2 [48.9; 63.6]
**RVSVi (ml/m^2^)**	46.3 [39.7; 54.2]	48.1 [44.2; 51.7]	48.9 [43.2; 59.5]	52.7 [42.4; 57.1]	51.9 [45.3; 61.9]	53.4 [52.5; 56.9]
**RVEDMM (g)**	34.3 [27.5; 4,8.1]	35.1 [30.5; 40.8]	51.9 [38.0; 67.4]	63.8 [50.5; 72.9]	74.4 [56.2; 99.7]	68.6 [61.7; 89.3]
**RVEDMMi (g/m^2^)**	48.9 [38.6; 56.4]	52.4 [42.9; 58.1]	44.7 [38.0; 52.8]	55.2 [47.0; 64.0]	49.1 [39.5; 56.7]	48.0 [42.4; 57.6]
**Neo-Ao RF (%)**	3.56 [2.35; 5.0]	4.39 [1.96; 10.89]	3.0 [1.9; 6.23]	3.17 [2.29; 7.64]	4.11 [2.72; 5.91]	5.08 [4.45; 7.41]

RVEDV, right ventricular end diastolic volume; RVEDVi, BSA indexed right ventricular end diastolic volume; RVESV, right ventricular end systolic volume; RVESVi, BSA indexed right ventricular end systolic volume; RVSV, right ventricular stroke volume; RVSVi, BSA indexed right ventricular stroke volume; RVEDMM, right ventricular end diastolic mass; RVEDMMi, indexed right ventricular end diastolic mass; Neo-Ao RF, Neo-aortic regurgitant fraction.

Values shown as median with interquartile range.

**Table 3:** Volumetrics for the LV remnant.

Parameters	Last examination
**LVEDV (ml)**	5.82 [2.97; 11.47]
**LVESV (ml)**	4.40 [2.51; 8.23]
**LVSV (ml)**	2.04 [1.12; 4.49]
**LVEF (%)**	32.94 [17.61; 46.89]
**LVEDVi (ml/ml^2^)**	4.90 [2.48; 7.67]
**LVESVi (ml/m^2^)**	3.97 [1.79; 6.70]
**LVSVi (ml/m^2^)**	1.59 [0.74; 5.16]
**LVEDMM (g)**	13.59 [7.74; 19.14]
**LVEDMMi (g/m^2^)**	9.93 [6.10; 12.83]

LVEDV, left ventricular end diastolic volume; LVEDVi, BSA indexed left ventricular end diastolic volume; LVESV, left ventricular end systolic volume; LVESVi, BSA indexed left ventricular end systolic volume; LVSV, leftventricular stroke volume; LVSVi, BSA indexed left ventricular stroke volume; LVEDMM, left ventricular end diastolic mass; LVEDMMi, indexed left ventricular end diastolic mass.

## 4 Discussion

Preserved RV function is essential for positive long-term outcome in patients with Fontan circulation [[8], [9], [10]]. This study assessed the influence of the presence and size of an LV remnant on systolic RV function, RV volumes and RV mass using longitudinal CMR imaging data. Our findings suggest that a rudimentary LV, however large, does not have a significant impact on RV volumetric parameters during medium-term follow up.

Previous studies that used echocardiography and cardiac catheterisation with the conductance method are in line with our findings. Wisler et al. could show by echocardiography, that LV size correlated significantly with poor RV systolic function in pre-Fontan patients, but this finding was no longer apparent after Fontan completion [11].

Schlangen et al. showed that anatomical subtype and LV size do not impact negatively on RV function early after Fontan completion [12]. However, in a more recent study, Cohen et al. could show that RV size and diastolic function were affected by the presence of a larger LV. Nevertheless, there was no difference in RV systolic function or transplantation-free survival on the basis of LV measures using echocardiography [2]. However, in both studies longitudinal data were not assessed.

In an early study, Walsh et al. could show that LV morphology, namely hypertrophy of the interventricular septum (IVS) caused by a larger LV remnant, may constitute a considerable risk factor during staged palliation [13]. However, outcome after Fontan completion was not analysed.

Few studies focused on outcome without specific RV analysis. It could be shown by Newland et al. that patients with a smaller LV experience more adverse events and a worse outcome than those with a larger LV [3]. However, they could not determine the exact cause of this, so reasons for this relationship could be manifold. Another large outcome study by Moon et al. suggested that patients with mitral stenosis and aortic atresia tend to have a greater rate of RV failure [13].RV dysfunction can also be the result of chronically elevated afterload, which can be caused by stenosis in the distal anastomosis or by recurrent coarctation [14]. This is not uncommon and of particular concern in right single ventricle patients, as the RV is more sensitive to afterload than the LV [15]. However, in our study, peak velocity at the distal anastomosis did not correlate significantly with RVEF.

Presence or absence of an LV remnant is not the only relevant factor when it comes to anatomical subtype in HLHS and RV function. Stamm et al. found that a dysplastic tricuspid valve is more frequent in patients with mitral stenosis as opposed to mitral atresia [16]. As tricuspid valve dysfunction is associated with an increased mortality risk [17], this anatomical feature might be of interest. Tricuspid regurgitation (TR) may also be relevant in the current context as the severity and progression of TR is shown to be associated with the severity of LV hypoplasia in HLHS [16].

### 4.1 Limitations

This study is retrospective and the number of patients who had a third CMR examination was lower than those who had two CMR studies. Furthermore, diastolic function was not assessed.

## 5 Conclusion

By analysing serial CMR examinations, it could be shown that the presence of an LV remnant does not impact negatively on RV size and function. Further longitudinal studies might focus on regional RV function.

## CRediT authorship contribution statement

**Abigail Burleigh:** Data curation, Formal analysis, Methodology, Writing – original draft. **Dominik Daniel Gabbert:** Supervision, Writing – review & editing. **Yujiro Ide:** Supervision, Writing – review & editing. **Anselm Uebing:** Supervision, Writing – review & editing. **Inga Voges:** Conceptualization, Formal analysis, Methodology, Supervision, Writing – original draft.

## Declaration of competing interest

The authors declare that they have no known competing interests or personal relationships that could have appeared to influence the work reported in this paper.
